# Molecular characterization of *Aeromonas hydrophila* detected in *Channa marulius* and *Sperata sarwari* sampled from rivers of Punjab in Pakistan

**DOI:** 10.1371/journal.pone.0297979

**Published:** 2024-03-29

**Authors:** Shahid Mahmood, Fayyaz Rasool, Muhammad Hafeez-ur-Rehman, Khalid Mahmood Anjum

**Affiliations:** 1 Department of Fisheries and Aquaculture, University of Veterinary and Animal Sciences, Lahore, Pakistan; 2 Department of Zoology, Faisalabad Campus, University of Education, Lahore, Pakistan; 3 Department of Wildlife and Ecology, University of Veterinary and Animal Sciences, Lahore, Pakistan; ICAR - Directorate of Coldwater Fisheries Research, INDIA

## Abstract

*Aeromonas hydrophila* is one of the major pathogenic bacteria responsible for causing severe outbreaks at fish farms and is also a major global public health concern. This bacterium harbors many virulence genes. The current study was designed to evaluate the antidrug and virulence potential of *A. hydrophila* by amplifying its antimicrobial resistance and virulence genes using PCR and examining their effects on fish tissues and organs. A total of 960 fish samples of *Channa marulius* and *Sperata sarwari* were collected from four sites of the rivers of the Punjab, Pakistan. *A. hydrophila* isolates were subjected to biochemical identification and detection of virulence and antimicrobial resistance (AMR) genes by PCR. We retrieved 181 (6.46%) *A. hydrophila* isolates from *C. marulius* and 177 (6.25%) isolates from *S. sarwari*. Amplification through PCR revealed the incidence of virulence genes in 95.7% of isolates in *C. marulius* and 94.4% in *S. sarwari*. Similarly, amplification through PCR also revealed occurrence of AMR genes in 87.1% of isolates in *C. marulius* and 83.9% in *S. sarwari*. Histopathological examination revealed congestion (5.2%) and hepatocyte necrosis (4.6%) in liver, lamellar fusion (3.3%) and the presence of bacterial colonies (3.7%) in gills, fin erosion (6%), and the presence of biofilms (3.5%) in tail fins of infected fish. Phylogenetic tree analysis of *16S rRNA* and *gyrB* gene of *A. hydrophila* revealed 100% and 97% similarity, respectively, with *16S rRNA* gene and *gyrB* of *A. hydrophila* isolated in previous studies. The results of antimicrobial susceptibility testing showed that all isolates demonstrated resistance to sulfamethoxazole, ampicillin, neomycin, and norfloxacin, while susceptibility to gentamicin, chloramphenicol, and tetracycline, and intermediate resistance was observed against cefotaxime. The results concluded that examined fish samples were markedly contaminated with virulent and multidrug strains of *A. hydrophila* which may be of a potential health risk. The study emphasizes the responsible antimicrobial use in aquaculture and the urgent need for effective strategies to control the spread of virulence and antimicrobial resistance genes in *A. hydrophila*.

## Introduction

The fast-growing aquaculture industry plays a vital role in global food security, offering high-quality protein, economic benefits, and jobs opportunities [1,2]. It also provides essential nutrients and a variety of food products [3,4]. In 2020, aquaculture contributed 122.6 million metric tons of aquatic products valued at USD 281.5 billion, with an annual growth rate of 6.7%. The current worldwide per capita fish consumption is 20.5 kg. Fish, in particular, is a cost-effective protein source, ranking second globally and accounting for 60% of protein intake [5]. It plays a crucial role in ensuring food security for the growing global population [6]. To meet demand, there has been a substantial increase in freshwater and marine fish production [7]. However, this expansion has intensified aquaculture systems, leading to water resource challenges and increased bacterial infections among cultivated aquatic organisms [8]. Despite these challenges, aquaculture remains a sustainable solution for global food security, helping mitigate food shortages driven by population growth [9].

Freshwater fish farming has been vital to the aquaculture industry’s growth, especially in Asia, providing food security, jobs, and economic benefits [10]. Various freshwater fish species like *Labeo rohita*, *Cirrhinus mrigala*, *Cyprinus carpio*, *Channa marulius*, *Sperata sarwari*, *Catla catla*, and *Pangasianodon hypophthalmus* have contributed to significant global commercial production [11]. These fish are prime candidates for aquaculture and have been widely cultivated [12,13]. However, it’s important to note that freshwater bodies, their primary habitat, also host the zoonotic pathogen *Aeromonas hydrophila*, which can infect fish, bivalves, amphibians, reptiles, and humans [14–16]. Two successful candidates in freshwater aquaculture are *Sperata sarwari* and *Channa marulius*, cultivated commercially in various regions, including Pakistan, India, Bangladesh, China, and Indonesia, where they are top producers [17].

*Channa marulius*, commonly known as the "Sole," thrives in diverse aquatic habitats like marshes, ponds, rivers, and rice fields, found in countries like China, India, Pakistan, Cambodia, and Thailand [18]. Belonging to the Channidae family, *C. marulius* is well-suited for intensive rearing systems due to its survival rate and rapid growth [19]. In Pakistan, it’s been introduced for commercial farming, standing out for its potential size of up to 30 kg [20,21]. In the Indus River, *Sperata sarwari* dominates the Bagridae catfishes, prized for its large size, valuable flesh, and low intramuscular bones [22]. Advances in aquaculture have enabled captive breeding for *S. sarwari* [23]. While most production relies on capturing, young *S. sarwari* occasionally enters the ornamental fish trade, easily distinguishable from other Bagridae catfish [24].

*Aeromonas hydrophila* is an emerging Gram-negative pathogen found in nature, belonging to the Aeromonadaceae family [25,26]. It is prevalent in aquatic environments, food sources, and mineral water bottles. This bacterium poses threats to both aquatic organisms, mainly fish, causing conditions like motile *Aeromonas* septicemia (MAS), ulcerative disease, and hemorrhagic septicemia [27], as well as humans, leading to wound infections, septicemia, and gastroenteritis. Factors contributing to its virulence include host susceptibility, environmental stressors, and virulence genes [28,29]. *A. hydrophila* is also a significant public health concern due to its potential for transferring virulence genes to humans. It can be found in various sources such as food, groundwater, wastewater, aquatic, and terrestrial animals [30,31]. Identification of *A. hydrophila* involves phenotypic methods and characterizing its *16S rRNA* gene and virulence genes [32,33]. Typically, its identification relies on the presence of virulence genes like the *aerolysin* gene (*aer*), *enterotoxin* gene (*ast*), *hemolysin A* gene (*hylA*), and *cytotoxic enterotoxin* gene (*act*) [33]. These virulence factors cause histopathological effects in fish [34]. The potential pathogens are associated with serious zoonotic infections [35].

The close interaction between naturally resistant bacteria in terrestrial and aquatic environments facilitates the rapid transfer of antimicrobial resistance (AMR) genes to pathogenic fish bacteria [36,37], making fish a vehicle for AMR bacteria and genes dissemination [38]. This results from fish farmers frequently using multiple antimicrobials to combat AMR bacteria [39], which, unfortunately, leads to an increase in antimicrobial-resistant (AMR) bacteria and their genes in aquaculture [40,41]. Addressing antimicrobial resistance within the One Health framework is crucial due to its interconnected impact on human, animal, and environmental health, requiring collaborative efforts for comprehensive solutions [42]. The emergence of AMR bacteria poses a significant challenge to public health [43,44], as they employ genetic strategies to resist antimicrobials [45]. Meanwhile, pathogenic bacterial diseases are a major cause of mass fish mortality in both cultured and farmed species [46], driven by virulence genes controlling factors like enzyme production [47], biofilm formation [48], immune system suppression, bloodstream infections [49], host-pathogen interactions [50], adaptation to various conditions [51], specificity to hosts [52], and epithelial cell lesions [53]. These factors directly impact nutrition, oxygen levels [54], growth phases [55], temperature [56], and pH in fish environments [57]. Regular monitoring and investigation of physicochemical parameters play a crucial role in controlling the prevalence of pathogenic bacteria [34,58].

The current study was designed to evaluate the antidrug and virulence potential of *A. hydrophila* by amplifying its antimicrobial resistance and virulence genes using PCR and examining their effects on fish tissues and organs.

## Materials and methods

### Ethical approval and, fish sampling

**Fig 1:**
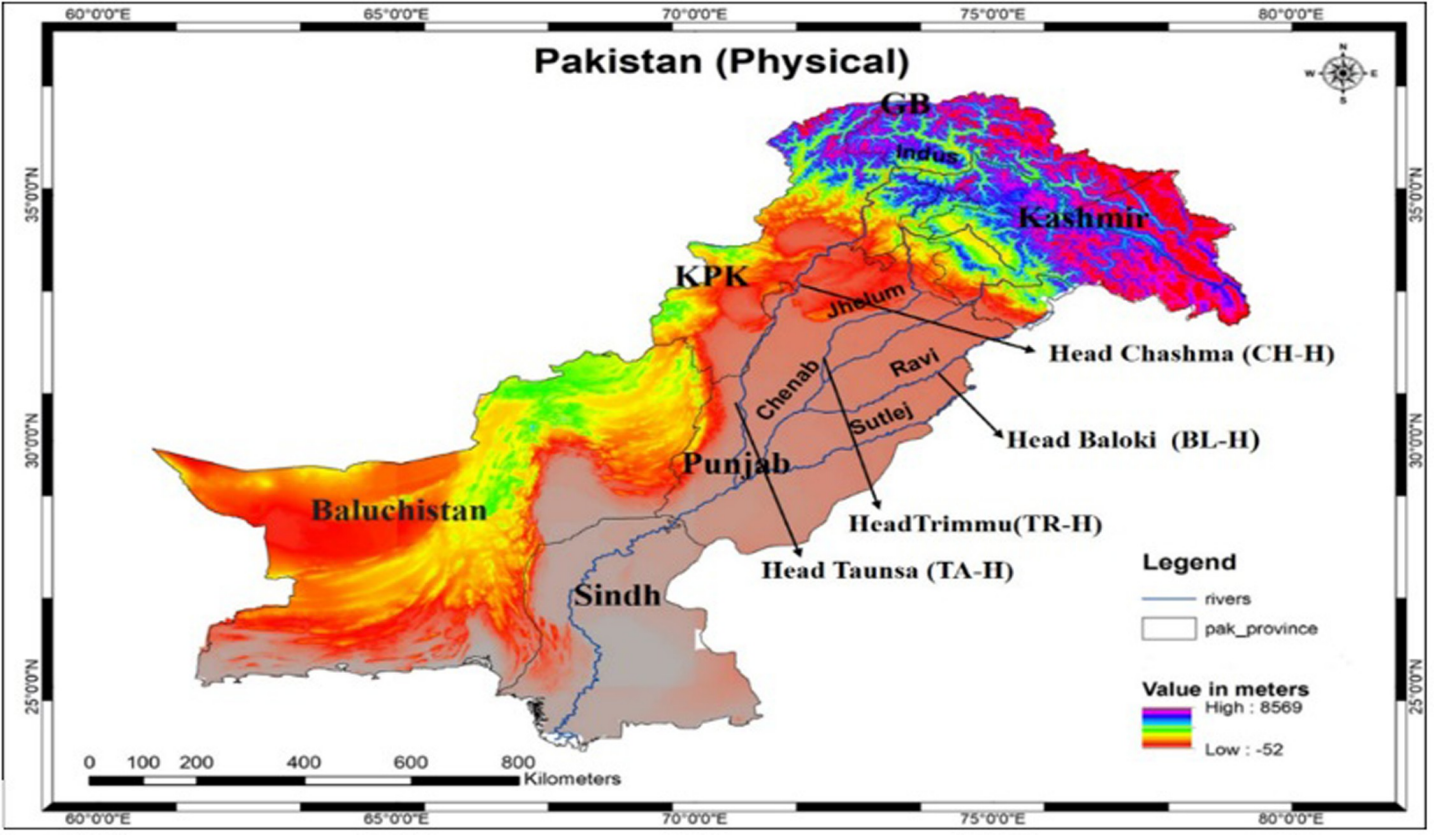
The sampling sites for the current study are depicted on the map.

All protocols and procedures were approved by the Guidelines for the Care and Use of Laboratory Animals Committee of the University of Veterinary and Animal Sciences, Lahore, Pakistan (DAS/358, 02-03-2023). A total of 960 fish samples (480 from each of *C. marulius* and *S. sarwari*) were collected using a nylon drag net from four selected sites: Head Baloki (BL-H), Head Taunsa (TA-H), Head Chashma (CH-H), and Head Trimmu (TR-H) of the riverine system of Punjab, Pakistan. Sampling was conducted from April 2022 to December 2022, categorized seasonally as 280 in summer, 120 in autumn, and 80 in winter. 120 fish samples of each species were collected from each sampling site. Soon after netting, the fish samples were measured for weight and length parameters outdoors. Water temperature of BL-H was measured as 25.42°C, 26.62°C at TR-H, 24.79°C at TA-H, and 22.98°C at CH-H. The sampling sites for the current study are depicted in Fig 1. The fish samples were placed in plastic containers with ice packs and transported directly (within 24 hours) to the laboratory of the Department of Zoology, University of Education, Faisalabad Campus, Pakistan.

### Isolation, phenotypic, morphological, and biochemical characterization of *Aeromonas hydrophila*

The collected fish was disinfected by rinsing with clean water and sodium hypochlorite following regulations and guidelines a recommended by Noga, [59]. The internal organs (skin, stomach, kidney, liver, intestine, spleen, and gills) of the collected fish were subjected to bacteriological examination. Swabs were randomly collected from suspected organs and were inoculated onto Trypticase soy agar (TSA LAB, UK) media by plate streaking method and were incubated at 37°C overnight according to the method described by Lima, and Muratori, [60,61]. A single colony from freshly obtained bacterial culture was inoculated onto Trypticase soy agar (TSA LAB, UK) media plates to obtain a pure culture of *A. hydrophila*, which was then incubated at 37°C for 24 h following the method recommended by Muratori, [61]. Pure culture of *A. hydrophila* was subjected to Gram-staining and viewed microscopically (Euromex, 100X). Colony morphology, culture, and microscopic characteristics of *A. hydrophila* were observed according to the protocol recommended by Muratori, and Xiao, [61,62]. The isolates of *A. hydrophila* were characterized by biochemical tests like indole, motility, oxidase, H_2_S production, catalase, and urease tests as for identification as previously performed by Fang, [63].

### DNA extraction

DNA was isolated using a Genomic DNA Purification Kit (Thermo Scientific, GeneJET, USA) and DNA samples were evaluated by gel electrophoresis on 1% agarose gel stained with ethidium bromide (Sigma-Aldrich E7637, USA) and utilizing a standard-sized molecular marker [1Kb DNA Ladder RTU (Ready-to-Use) GeneDireX, Taiwan]. Isolated DNA was stored at -20°C for further use.

### Amplification, sequencing, and phylogenetic tree analysis of *16S rRNA* and *gyrB* gene of *A. hydrophila*

One microliter of template DNA was added into a total of 25 μl reaction solution for PCR containing two primers of *16S rRNA*; 1 μl forward primer (27F): AGAGTTTGATCCTGGCTCAG, 1 μl reverse primer (1492R): GGTTACCTTGTTACGACTT, 10 μl PCR-grade water, and 12 μl GoTaq Green Master Mix (Promega, USA) (**Table 1**). Similarly, *gyrB* gene was also amplified by species-specific primers. PCR products were electrophoresed in 1% agarose gel stained with ethidium bromide (Sigma-Aldrich E7637, USA) and utilizing a standard-sized molecular marker (1Kb DNA Ladder RTU, GeneDireX). PCR products revealing the thickest bands were sequenced by Sanger’s method at BGI Hong Kong Co. Ltd. China. The obtained sequences were analyzed and compared for taxonomic identification using National Centre for Biotechnology Information-Basic Local Alignment Search Tool (NCBI-BLAST), and subsequently, submitted to the GenBank^®^ database. To determine the phylogenetic relationship of *A. hydrophila*, a phylogenetic tree analysis was conducted on the *16S rRNA* and *gyrB* genes of *A. hydrophila*. This analysis employed the bootstrap method with 1,000 bootstrap replications, and it was carried out using MEGA 11.0 (Molecular Evolutionary Genetic Analysis), as described by Chen, [64].

**Table 1:** Conditions for amplification of *16S rRNA*, *gyrB*, virulence, and AMR genes of *A. hydrophila*.

Gene Class	Gene Name	Primer sequence (5–3)	Target bp size	Annealing Temperature	Accession Number	References
**rrs gene**	*16S rRNA*	F-AGAGTTTGATCCTGGCTCAG	1503	52°C for 1min	OR509789; OR509790 OR509791; OR509792 OR509793; OR509794	[65]
		R-ACGGCTACCTTGTTACGACTT				
**Antimicrobial Resistance genes (AMR)**	*gyrB*	F-GAGGACTACAGCAAGAAGGCCA	1124	55°C for 90 s	OQ699127	[66]
		R-GACTTGGCCTTCTTGCTGTAGTC				
	*tetA*	F-GCTACATCCTGCTTGCCTTC	813	55°C for 1min	OR061081	[67]
		R-CATAGATCGCCGTGAAGAGG				
	*blaTEM*	F-CATTTCCGTGTCGCCCTTATTC	873	55°C for 90 s	OQ726106	
		R-CGTTCATCCATAGTTGCCTGAC				
	*qnrA*	F-ATTTCTCACGCCAGGATTTG	654	60°C for 1 min	OQ729990	[68]
		R-GATCGGCAAAGGTTAGGTCA				
	*qnrB*	F-GGMATHGAAATTCGCCACTG	642	53°C for 30 s	OR515644	[69]
		R-TTTGCYGYYCGCCAGTCGAAC				
	*sul1*	F-CGGCGTGGGCTACCTGAACG	444	55°C for 30 s	OR515645	[70]
		R-GCCGATCGCGTGAAGTTCCG				
	*sul3*	F-AGATGTGATTGATTTGGGAGC	444	54.2°C for 30 s	OR061080	[68]
		R-TAGTTGTTTCTGGATTAGAGCCT				
**Virulence gene**	*hylA*	F-GGCAAACAGCGAAACAAATACC	585	55.5°C for 30 s	OR515643	[71]
		R-CTCAGCGGGCTAATACGGTTTA				
	*aerA*	F-GTCACCTTCTCGCTCAGGC	417	55°C for 30 s	OR515642	[72]
		R-TGATTCCCGAAGGCACTCCC				
	*act*	F-GAGAAGGTGACCACCAAGAACA	675	58°C for 30 s	OR515641	
		R-AACTGACATCGGCCTTGAACTC				

### Molecular identification of virulence and antimicrobial resistance (AMR) genes of *A. hydrophila*

Virulence genes of *A. hydrophila* (including *hemolysin* (*hylA*), *aerolysin* (*aerA*), and *cytotoxic enterotoxin* (*act*)) and antimicrobial resistance genes (such as *sul1*, *sul3*, *qnrA*, *qnrB*, *blaTEM*, and *tetA*) of *A. hydrophila* were identified through PCR analysis using species-specific primers (Macrogen, Korea) and were compared with a standard-sized molecular marker DNA ladder (**Table 1**). A total of 25 μl of PCR reaction solution, comprising 1 μl of template DNA, 1 μl forward primer, 1 μl reverse primer, 10 μl PCR-grade water, and 12 μl GoTaq Green Master Mix (Promega, USA), was utilized for the detection of the AMR genes in *A. hydrophila* (**Table 1**). Amplified PCR products were analyzed on 1% agarose gel stained with ethidium bromide (Sigma-Aldrich E7637, USA) and utilizing a standard-sized molecular marker (1Kb DNA Ladder RTU, GeneDireX). PCR products revealing the thickest bands were sequenced by Sanger’s method at BGI Hong Kong Co. Ltd., China as previously analyzed by Wang, [73].

### Minimal inhibitory concentration (MIC) and antimicrobial susceptibility testing of *A. hydrophila*

*A. hydrophila* isolates were subjected to microtiter plates and Kirby Bauer disc diffusion method for antimicrobial sensitivity testing on Mueller-Hinton agar plates according to the method carried out by Bauer, [74] using antimicrobials norfloxacin, streptomycin, gentamicin, chloramphenicol, ciprofloxacin, doxycycline, ampicillin, flumequine, neomycin, tetracycline, sulfamethoxazole, and cefotaxime. The plates were incubated for twenty-four hours at 37°C. Diameter of the inhibition zone were measured and interpreted to classify bacteria as resistant, moderately susceptible, and susceptible according to clinical and laboratory standards institute (CLSI), [75].

### Histopathological effect of *A. hydrophila*

Tissue samples were collected from the liver, stomach, spleen, and small intestine of infected *C. marulius* and *S. sarwari*. These collected tissue specimens were disinfected and preserved in a 10% neutral buffered formalin solution with a 1:10 ratio (formalin and distilled water, respectively) in plastic sample containers, labeled against each respective tissue specimen. The preserved tissue samples were submitted to the laboratory of the Department of Pathology, City Campus, University of Veterinary and Animal Sciences (UVAS) Lahore, and examined for histopathological changes due to *A. hydrophila* infection, specifically motile *Aeromonas* septicemia (MAS). The obtained slides were viewed under a light microscope (Euromex 100X, Netherlands) to observe histopathological changes caused by *A. hydrophila* and stored for future use.

### Statistical analysis

Chi-square test of independence was applied in comparing the prevalence/occurrence of *A. hydrophila* with respect to sampling site, fish sex, season, and organs. Descriptive statistics such as proportions and frequency were employed in summarizing the data.

## Results

### Physicochemical parameters, analysis of weight and length of *C. marulius* and *S. sarwari*

Maximum and minimum temperature was recorded as 26.62°C (TR-H) and 22.98°C (CH-H) respectively. Maximum and minimum pH was recorded as 8.23 (CH-H) and 7.18 (TR-H) respectively. Samples of *S. sarwari* collected from CH-H showed maximum weight (307 g) and minimum by fish collected from TR-H (303.8 g) while maximum length (27.4 cm) was shown by samples of *S. sarwari* collected from TR-H and minimum length (25.6 cm) by fish collected from BL-H. Similarly, samples of *C. marulius* collected from CH-H showed maximum weight (175 g) and minimum by fish collected from BL-H (151.2 g) while maximum length (34.2 cm) was shown by samples of *C. marulius* collected from CH-H and minimum length (27.4 cm) by fish collected from BL-H. Results of physicochemical parameters, weight and length of *C. marulius* and *S. sarwari* are shown in **S1 Table**.

### Isolation, phenotypic and biochemical characterization

We collected swabs from the organs of 480 fish samples of each of *S. sarwari* and *C. marulius*. We isolated *A. hydrophila* by direct plating on TSA plates. We recovered *A. hydrophila* in 31 fish samples of *C. marulius* and 30 of *S. sarwari* collected from all sampling sites. Phenotypic characterization of *A. hydrophila* showed rod-shaped, round, smooth, and grayish-white colored colonies on TSA media plates. Biochemical characterization of *A. hydrophila* isolates revealed it as motile, Gram-negative, rod-shaped, and facultatively anaerobic bacterium bearing Peritrichous flagella, by biochemical tests. All the isolates of *A. hydrophila* were found positive against catalase, oxidase, glucose, sucrose, lactose, urease, indole, and H_2_S production tests represented in **S2 Table.**

### Prevalence of *A. hydrophila*

Overall prevalence of *A. hydrophila* was recorded as 6.35% in fish samples of both fish species. The maximum prevalence of *A. hydrophila*, 6.46% was observed in the intestine of infected *C. marulius* while, the minimum prevalence, 4.17% was noted in gills of infected *S. sarwari* (**Table 2**). Overall *A. hydrophila* infected 15 fish samples (12.5%) of *S. sarwari* collected from BL-H while, the minimum infection rate, 1.67% was observed in *C. marulius* collected from CH-H. Among the fish, *A. hydrophila* infected 9.78% of males in *C. marulius* and 4.7% of females in *S. sarwari*. Furthermore, *A. hydrophila* infected 6.43% and 5.71% of fish samples of *S. sarwari* and *C. marulius* respectively during the summer while 5% and 6.25% of *S. sarwari* and *C. marulius* respectively during the winter **Table 3**.

**Table 2:** Prevalence of *A. hydrophila* with respect to fish organs.

Fish Species	Fish Organs						
	Skin	Liver	Intestine	Stomach	Gills	Kidney	Spleen
***S. sarwari***	21 (4.37%)	26 (5.42%)	28 (5.83%)	30 (6.25%)	20 (4.17%)	29 (6.04%)	23 (4.79%)
***C. marulius***	24 (5%)	25 (5.21%)	31 (6.46%)	29 (6.04%)	23 (4.79%)	27 (5.62%)	22 (4.58%)

**Table 3:** Prevalence of *A. hydrophila* with respect to sampling sites, sex, seasons and overall prevalence.

Fish Species	Sampling sites				SEX		SEASONS			Overall prevalence
	Head Baloki (BL-H)	Head Trimmu (TR-H)	Head Taunsa (TA-H)	Head Chashma (CH-H)	Male	Female	Summer	Autumn	Winter	
***S. sarwari***	15 (12.5%)	8 (6.67%)	4 (3.33%)	3 (2.5%)	18 (8%)	12 (4.7%)	18 (6.43%)	8 (6.67%)	4 (5%)	30 (6.25%)
***C. marulius***	13 (10.83%)	11 (9.17%)	5 (4.17%)	2 (1.67%)	22 (9.78%)	9 (3.53%)	16 (5.71%)	10 (8.33%)	5 (6.25%)	31 (6.40%)

### Occurrence of virulence and antimicrobial resistance genes of *A. hydrophila*

Virulence genes (*aerA*, *hylA*, and *act*) and antimicrobial resistance genes (*sul1*, *sul3*, *qnrA*, *qnrB*, *tetA*, and *blaTEM*) of *A. hydrophila* were amplified by PCR. Among all the AMR genes, maximum occurrence, 6.04% of *tetA* gene was recorded in *A. hydrophila* isolates isolated from *C. marulius* (**Table 4**). Similarly, among all the virulence genes, maximum occurrence, 6.46% of *aerA* gene was recorded in *A. hydrophila* isolates isolated from *C. marulius*. The chi-square test of independence showed insignificant difference (P>0.05) in occurrence of antimicrobial resistance (AMR) genes **Table 5**.

**Table 4:** Occurrence of antimicrobial resistance (AMR) genes, *gyrB*, and *16S rRNA* gene of *A. hydrophila*.

Fish Species	*tetA*	*blaTEM*	*qnrA*	*qnrB*	*sul1*	*sul3*	*aerA*	*hylA*	*act*	*gyrB*	*16S rRNA*
***S. sarwari***	22 (4.58%)	25 (5.21%)	28 (5.83%)	23 (4.79%)	28 (5.83%)	24 (5%)	30 (6.25%)	28 (5.83%)	27 (5.62%)	27 (5.62%)	30 (6.25%)
***C. marulius***	29 (6.04%)	19 (3.96%)	27 (5.62%)	21 (4.37%)	26 (5.42%)	21 (4.37%)	31 (6.46%)	30 (6.25%)	28 (5.83%)	31 (6.46%)	31 (6.46%)

**Table 5:** The results of the chi-square test of independence show the X^2^-value and P-value in relation to the parameters.

Parameter	*S. sarwari*		*C. marulius*	
	Chi-squared value	p-value	Chi-squared value	p-value
**Organs**	35.00	0.243 ^ns^	108.5	0.628^ns^
**Bacterial Species**	20.00	0.220 ^ns^	88.3	0.158^ns^
**Sampling Sites**	12.00	0.213 ^ns^	37.3	0.408^ns^
**Fish Sex**	2.00	0.157 ^ns^	8.0	0.433^ns^
**Seasons**	6.00	0.199 ^ns^	30.0	0.268^ns^
**Occurrence of AMR Genes**	8.00	2.38 ^ns^	36.0	0.607^ns^

Note; ns indicate Non-significant.

### Multiple-drug resistance (MDR) and antimicrobial susceptibility testing

Antimicrobial susceptibility testing was performed on a total of 30 *S. sarwari* and 31 *C. marulius* isolates of *A. hydrophila*. All the isolates of *A. hydrophila* demonstrated resistance to amoxicillin, ampicillin, sulfamethoxazole, erythromycin, flumequine, ciprofloxacin, neomycin, and norfloxacin. In contrast, *A. hydrophila* isolates demonstrated susceptible to gentamicin, doxycycline, chloramphenicol, and tetracycline, with intermediate resistance observed against cefotaxime and streptomycin shown in **Tables 6–8.**

**Table 6:** Results of antimicrobial susceptibility of *A. hydrophila*.

Antibiotics	Concentration (μg)	Class of Antimicrobial	Susceptible	Intermediate	Resistant	MIC_90_ (μg /ml)	MIC_50_ (μg /ml)
**AMX**	25	Penicillin	0	0	100%	>128	>64
**AMP**	10	Penicillin	0	0	100%	>64	>32
**CTX**	5	Cephalosporin	0	50%	0	<32	<16
**C**	30	Miscellaneous Antibiotics	100%	0	0	1	0.5
**CIP**	5	Fluoroquinolones	25%	0	75%	>32	>16
**DO**	30	Tetracycline	100%	0	0	1	0.5
**E**	15	Miscellaneous Antibiotics	0	0	100%	16	8
**FLU**	30	Quinolones	0%	0	100%	>128	>64
**GM**	10	Aminoglycosides	100%	0	0	4	2
**N**	30	Aminoglycosides	0	0	100%	>128	>64
**NOR**	10	Fluoroquinolones	0	0	100%	>64	>32
**S**	10	Aminoglycosides	0	50%	0	4	2
**SXT**	25	Sulfonamides	0	0	100%	>64	>32
**T**	10	Tetracycline	100%	0	0	<4	<2

Note: AMX indicates Amoxicillin, AMP Ampicillin, CTX Cefotaxime, C Chloramphenicol, CIP Ciprofloxacin, DO Doxycycline, E Erythromycin, FLU Flumequine, GM Gentamicin, N Neomycin, NOR Norfloxacin, S Streptomycin, SXT Sulfamethoxazole and, T Tetracycline.

**Table 7:** MDR profile for *A. hydrophila* isolated from indus riverine fish Punjab-Pakistan.

Antibiotic Combination	No of Isolates	No of Antibiotic Resistance	%age
**AMY**	1	1	1.6
**AMP, E**	1	2	11.5
**FLU, N**	1	2	
**N, NOR**	2	2	
**AMY, SXT**	3	2	
**AMY, AMP, E**	1	3	8.2
**FLU, N, NOR**	1	3	
**SXT, E, AMP**	1	3	
**AMY, NOR, N**	2	3	
**AMY, AMP, E, FLU**	1	4	14.8
**N, NOR, SXT, AMY**	2	4	
**AMP, E, FLU, N**	4	4	
**NOR, SXT, AMY, E**	2	4	
**AMY, AMP, E, FLU, N**	1	5	18
**AMP, E, FLU, N, NOR**	2	5	
**E, FLU, N, NOR, SXT**	1	5	
**FLU, N, NOR, SXT, AMY**	1	5	
**N, NOR, SXT, AMY, AMP**	3	5	
**NOR, SXT, AMY, AMP, E**	2	5	
**SXT, E, AMP, AMY, FLU**	1	5	
**AMY, AMP, E, FLU, N, NOR**	2	6	36
**AMY, AMP, E, FLU, N, SXT**	1	6	
**AMY, AMP, E, FLU, NOR, SXT**	1	6	
**AMY, AMP, E, N, NOR, SXT**	6	6	
**AMY, AMP, FLU, N, NOR, SXT**	1	6	
**AMY, E, FLU, N, NOR, SXT**	8	6	
**AMP, E, FLU, N, NOR, SXT**	3	6	
**AMY, AMP, E, FLU, N, NOR, SXT**	6	7	9.8
**TOTAL**	61	121	100%

Note. AMX indicates Amoxicillin, AMP Ampicillin, CTX Cefotaxime, C Chloramphenicol, CIP Ciprofloxacin, DO Doxycycline, E Erythromycin, FLU Flumequine, GM Gentamicin, N Neomycin, NOR Norfloxacin, S Streptomycin, SXT Sulfamethoxazole and, T Tetracycline.

**Table 8:** Resistance genes profile for *A. hydrophila* isolated from indus riverine fish Punjab-Pakistan.

Resistance gene	No of Isolates	No of Resistance genes	%age
***tetA*, *blaTEM*, *qnrA*, *qnrB***	1	4	24.6
***blaTEM*, *qnrA*, *qnrB*, *Sul1***	1	4	
***qnrA*, *qnrB*, *Sul1*, *Sul3***	6	4	
***qnrB*, *Sul1*, *Sul3*, *tetA***	1	4	
***Sul1*, *Sul3*, *tetA*, *blaTEM***	4	4	
***Sul3*, *tetA*, *blaTEM*, *qnrA***	2	4	
***tetA*, *blaTEM*, *qnrA*, *qnrB*, *Sul1***	2	5	60.7
***blaTEM*, *qnrA*, *qnrB*, *Sul1*,*sul3***	8	5	
***qnrA*, *qnrB*, *Sul1*, *Sul3*, *tetA***	5	5	
***qnrB*, *Sul1*, *Sul3*, *tetA*, *blaTEM***	6	5	
***Sul1*, *Sul3*, *tetA*, *blaTEM*, *qnrA***	6	5	
***Sul3*, *tetA*, *blaTEM*, *qnrA*, *qnrB***	10	5	
***tetA*, *blaTEM*, *qnrA*, *qnrB*, *Sul1*, *sul3***	9	6	14.7
**Total**	61	60	100%

Note; tetA indicate tetracycline, β*-*lactamase *blaTEM*, Quinolones *qnrA*, *qnrB* and sulfonamide resistance gene *Sul1*, *sul3*.

### Phylogenetic tree analysis

**Fig 2:**
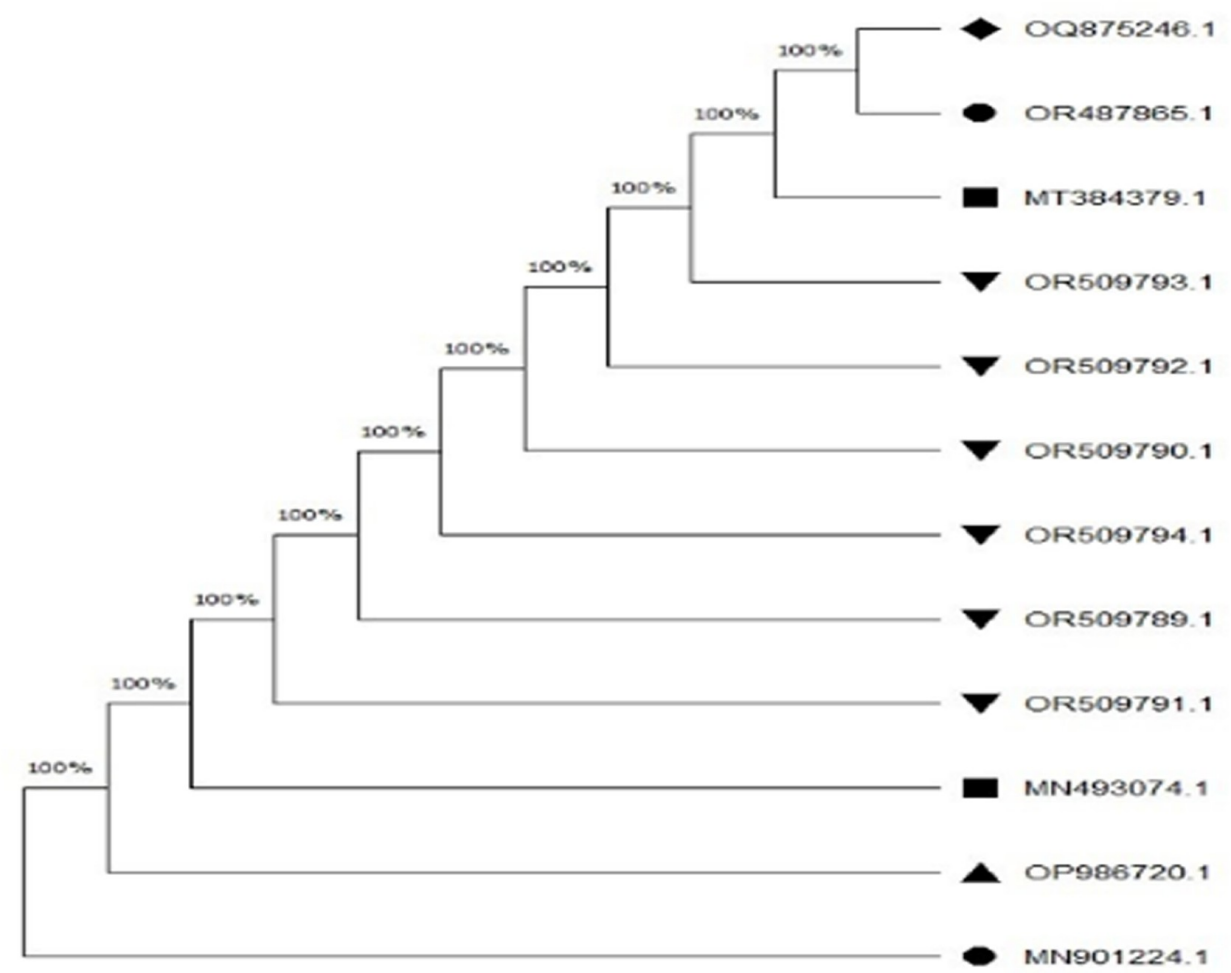
Phylogenetic tree analysis of *16S rRNA* gene of *A. hydrophila*.

**Fig 3:**
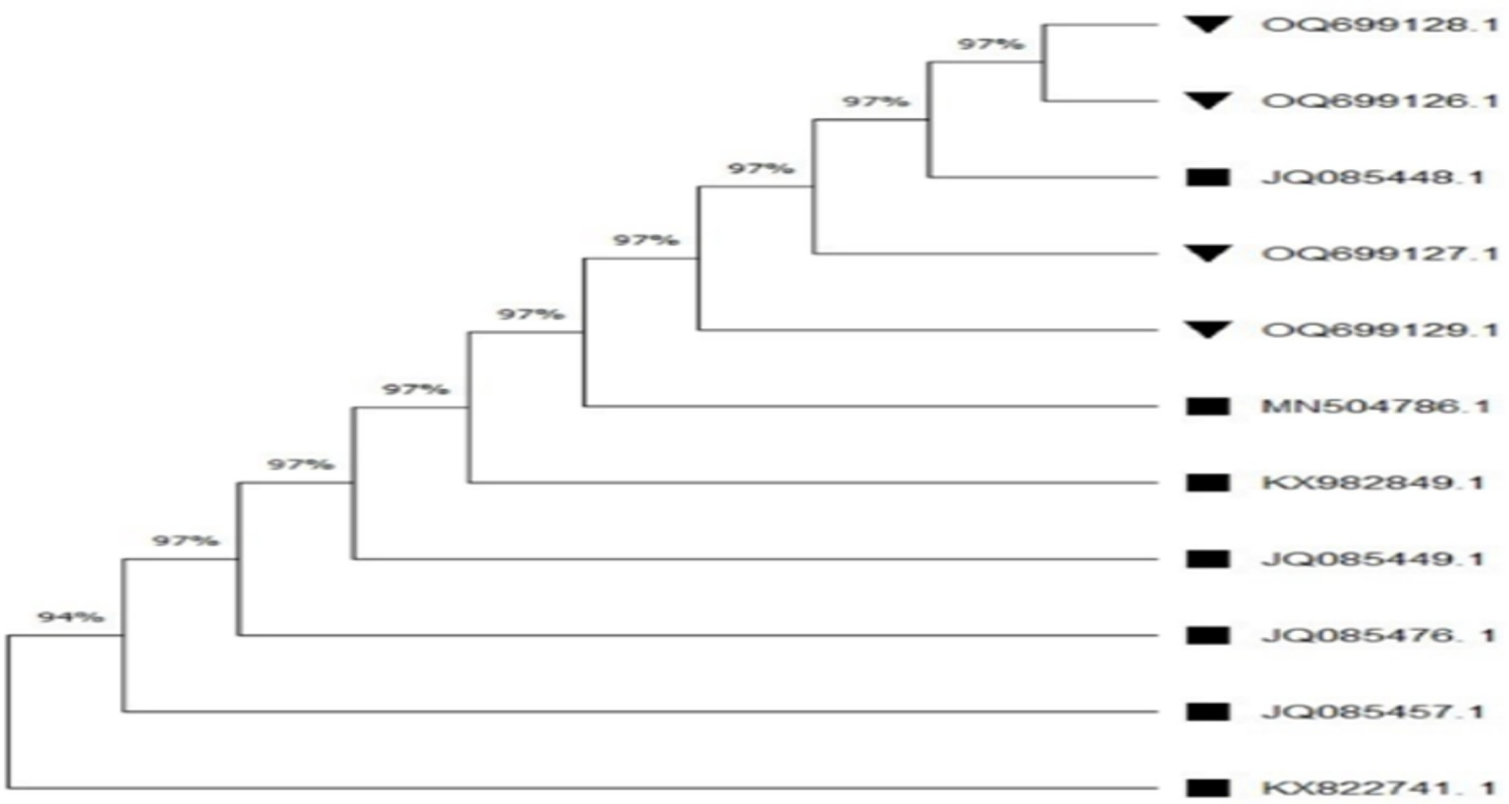
Phylogenetic tree analysis of *gyrB* gene of *A. hydrophila*.

Phylogenetic tree of *16S rRNA* gene *A. hydrophila* revealed 100% similarity among all the *A. hydrophila* strains isolated in the current study, as well as with strains isolated in earlier studies (**Fig 2**). Furthermore, phylogenetic tree analysis of *gyrB* gene of *A. hydrophila* revealed 97% similarity among all the *A. hydrophila* strains isolated in the current study, as well as with strains previously isolated (**Fig 3**).

### Histopathological effect of *A. hydrophila*

**Fig 4:**
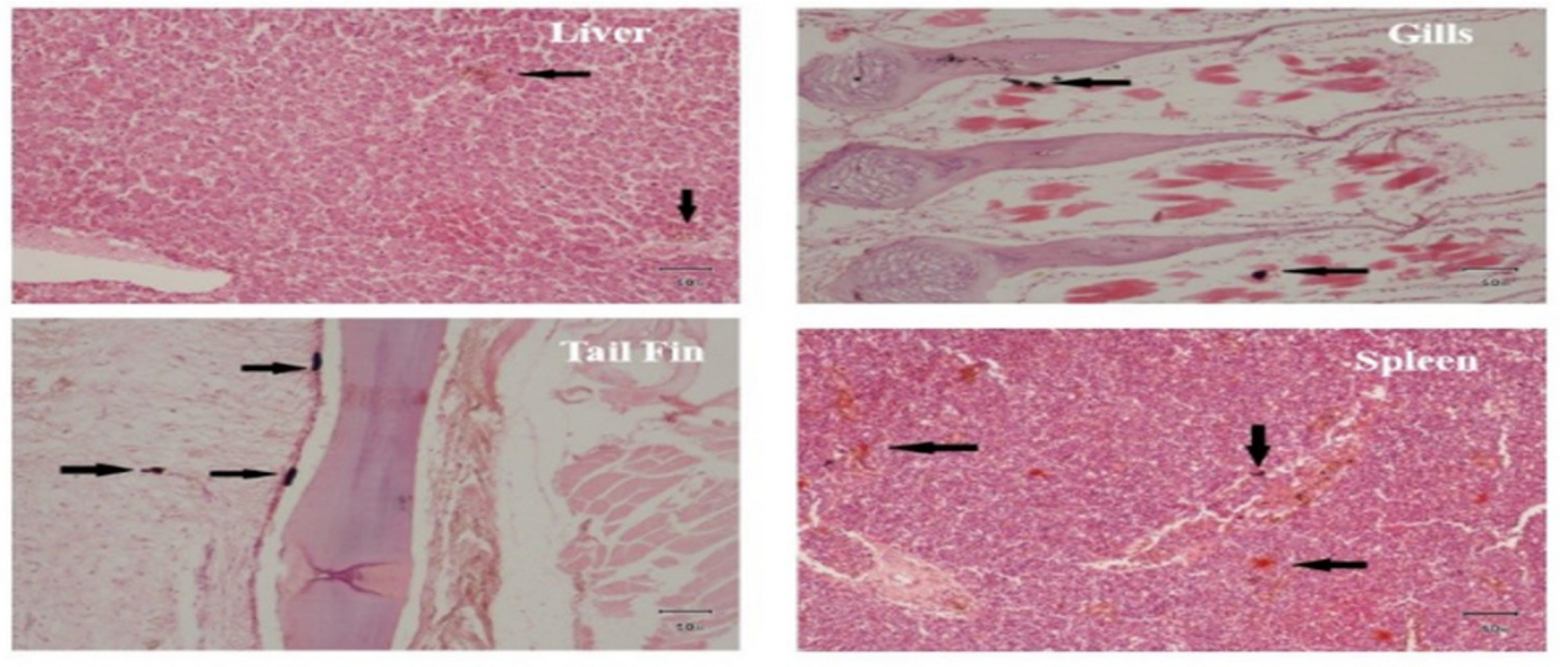
Histopathological impact of *A. hydrophila* on tissues of infected fish samples.

Histopathological examination revealed various abnormalities in the infected fish. In the liver, findings included congestion (5.2%), hepatocyte necrosis (4.6%), granuloma formation (4.3%), and inflammation (5%). The gills exhibited epithelial hyperplasia (3.5%), lamellar fusion (3.3%), edema (3%), and the presence of *A. hydrophila* colonies (3.7%). Tail fins displayed issues such as fin erosion (6%), hemorrhage (6.2%), loss of fin rays (4.8%), and the presence of biofilms (3.5%). *A. hydrophila* infection also led to fibrosis (4%), abscess formation (3.7%), fatty degeneration (3.5%), and the infiltration of inflammatory cells (4.7%) in spleen (**Fig 4**).

## Discussion

Fish is one of the most important sources of food that provides easy digestion, high palatability, and high nutritional value. However, it is also considered an important vehicle for many types of pathogens, raising public health concerns. The prevalence of *A. hydrophila* is directly proportional to an increase in temperature, but there is no association between its prevalence and the weight and length of the fish. In the current study, overall 61 *A. hydrophila* (6.3%) were recovered in both fish species, *C. marulius* and *S. sarwari*. The intestine and stomach were reported as the organs with a high prevalence of *A. hydrophila*, as 31 isolates of intestine (6.5%) from *C. marulius* and 30 isolates of stomach (6.2%) from *S. sarwari* showed the maximum prevalence. An increase in temperature during the summer also favors a higher prevalence, as 34 isolates were recorded during this season (3.5%). *A. hydrophila* affects males more than females, as the maximum prevalence was recorded in 40 (4.2%) male fish samples from both species. Additionally, the highest prevalence was found in 28 fish samples (2.9%) at Head Baloki (BL-H) in Kasur.

Phenotypic characterization in the current study confirmed *A. hydrophila* isolates as circular, smooth, Gram-negative, rod-shaped, motile, and facultatively anaerobic bacteria bearing peritrichous flagella. Biochemical tests showed that all *A. hydrophila* isolates were positive for catalase, oxidase, glucose, sucrose, lactose, urease, indole, and H_2_S production tests. In a previous study, Wamala, [76] identified *A. hydrophila* isolates as Gram-negative, motile, and positive in catalase, oxidase, and indole production tests in Uganda. However, they observed negative results in urease and H_2_S production tests, which contradicted our findings. Li, [77] in a study conducted in China, observed positive results in glucose and H_2_S production tests but negative results in the urease test, again differing from our findings.

In the current study, we detected three virulence genes, namely *aerolysin* (*aerA*), *hemolysin* (*hylA*), and *cytotoxic enterotoxin* (*act*) genes, in *A. hydrophila* isolates recovered from a total of 31 samples (6.25%) of *C. marulius* and 30 samples (6.46%) of *S. sarwari*. Specifically, we observed *aerA* gene in 31 isolates (6.45%), *hylA* gene in 30 isolates (6.25%), and *act* gene in 28 isolates (5.83%) of infected *C. marulius*. Similarly, we recorded *aerA* gene in 30 isolates (6.25%), *hylA* gene in 28 isolates (5.8%), and *act* gene in 27 isolates (5.6%) of infected *S. sarwari*. In a recent study, Morshdy, [78] recovered *A. hydrophila* in 20% of catfish samples in Egypt. They also detected *aerolysin* and *hemolysin* genes in 25% and 75% of retail fish samples, respectively. The main reason behind the high prevalence of *A. hydrophila* was contamination caused by marketing and transportation. Similarly, El-Hossary, [79] detected *aerolysin* (*aerA*) and *hemolysin* (*hylA*) genes in *A. hydrophila* isolated from infected Nile tilapia (*Oreochromis niloticus*) collected from local fish markets in Egypt. They found *A. hydrophila* in 28.8% of market fish samples. The variations in the prevalence of *A. hydrophila* could be attributed to various factors, including sampling conditions (such as location and time), post-capture contamination, fish species, handling, water type, geographic location, manipulations during capture, storage, marketing, and transportation. Moreover, Thaotumpitak, [80] recovered 15 isolates (5.39%) of *A. hydrophila* in hybrid tilapia collected from cage culture in Thailand. They also detected *aerolysin* (*aerA*) and *hemolysin* (*hylA*) genes in *A. hydrophila* in infected hybrid tilapia. Additionally, Suresh and Pillai, [29] recovered *A. hydrophila* from 27% of samples of Indian major carps (*Cirrhinus mrigala*, *Labeo rohita*, and *Catla catla*) in India. They identified ten virulence genes, including *aerolysin* (*aerA*), *hemolysin* (*hylA*), and *cytotoxic enterotoxin* (*act*) genes, in the infected fish. The variation in the prevalence of *A. hydrophila* could be attributed to stress, which allows the opportunistic pathogen *A. hydrophila* to cause infections.

As an opportunistic pathogen, *A. hydrophila* infects fish under conditions of stress, high temperature, low water quality, high organic content, and stocking density. In a recent study, Abdella, [28] detected 312 virulence genes in *A. hydrophila* strains, including *aerA*, *hylA*, and *act* genes in Egypt. In another study, by Nhinh, [81] 46.4% of *A. hydrophila* isolates were recovered from 506 diseased (moribund) tilapia, carps (common carp and grass carp), and channel catfish of Vietnam. They also detected the *aerA* gene in 80.1% of cases and the *act* gene in 80.5% of cases. Similarly, Saleh, [82] recovered 53.4% (187/350) of *A. hydrophila* isolates from infected Nile tilapia in Egypt. They detected the *act* and *aerA* genes in virulent *A. hydrophila* strains. In a similar study, Ahmed, [83] found *A. hydrophila* in 34 isolates (7.1%) isolated from Nile tilapia (*O. niloticus*) and *Mugil cephalus* in Egypt. They also identified four virulence genes, including *hly*, *aer*, and *act* genes, in infected fish samples. Additionally, Azzam-Sayuti, [84] recovered 20% of *A. hydrophila* isolated from 270 healthy cultured *Clarias batrachus*, *P. hypophthalmus*, and *O. niloticus* in Malaysia. They detected eight virulence genes, including *aerA*, *hylA*, and *act* genes. Moreover, Abu-Elala, [85] recovered 20 out of 24 (83.3%) *A. hydrophila* isolates from infected fish in Egypt. They detected 45.45% of virulence genes in *A. hydrophila* isolates, including the *aer* and *act* genes. Similarly, Roges, [86] reported a 92.7% occurrence of virulence genes in 110 *A. hydrophila* isolates isolated from fish, animals, and humans, including the *act*, *aer*, and *hyl* genes in Brazil. The major reasons behind these significant variations in results may include contaminated water, severe environmental conditions, bacterial strains, and low water quality parameters.

*A. hydrophila* is a multiple antimicrobial-resistant bacterium and one of the most significant pathogens in fish, causing *Aeromonas* septicemia (MAS) in various freshwater fish species. Its antimicrobial resistance against multiple drugs has made it a global health risk. In the current study, we identified the presence of *blaTEM*, *sul1*, *sul3*, *qnrA*, *qnrB*, and *tetA* genes in *A. hydrophila* isolated from both *C. marulius* and *S. sarwari*. Specifically, we recorded a 6.46% prevalence of the *tetA* gene, 6.25% for *blaTEM*, 5.83% for *sul1*, 5.42% for *sul3*, 5% for *qnrA*, and 4.17% for *qnrB* gene in 31, 30, 28, 26, 24, and 20 samples of infected *C. marulius*, respectively. Similarly, in *S. sarwari*, we recorded a 6.25% prevalence of the *tetA* gene, 6.04% for *blaTEM*, 5.21% for *sul1*, 4.79% for *sul3*, 4.58% for *qnrA*, and 4.37% for *qnrB* gene in 30, 29, 25, 23, 22, and 21 samples of infected fish, respectively.

We observed that all *A. hydrophila* isolates were resistant to amoxicillin, ampicillin, sulfamethoxazole, neomycin, and norfloxacin, while they were susceptible to gentamicin, chloramphenicol, and tetracycline. Additionally, they showed intermediate resistance to cefotaxime. In a recent study, Eid, [87] reported a 53.85% prevalence of *A. hydrophila* collected from Mediterranean seawater in Egypt. They identified *sul1*, *blaTEM*, and *tetA* genes in *A. hydrophila* isolated from *M. cephalus* (striped mullet) in Egypt and also detected the *act* gene in antimicrobial-resistant *A. hydrophila*. These isolates were resistant to β-lactams and sulfonamides (100%), oxytetracycline (90%), and streptomycin (62.22%), but completely susceptible to cefotaxime. In a recent study, Thaotumpitak, [80] identified six antimicrobial resistance genes in *A. hydrophila* isolated from hybrid red tilapia cultured in cages in Thailand, including *blaTEM*, *sul1*, *sul3*, *qnrA*, *qnrB*, and *tetA*. All *A. hydrophila* isolates were resistant to ampicillin, oxytetracycline, tetracycline, trimethoprim, and oxolinic acid. Similarly, Fauzi, [88] reported the presence of drug resistance genes in *A. hydrophila* isolated from freshwater fish in Malaysia. They identified *sul1*, *blaTEM*, and *tetA* genes in *A. hydrophila*. These isolates were resistant to ampicillin, kanamycin, nalidixic acid, neomycin, oxytetracycline, streptomycin, tetracycline, and sulfamethoxazole. Additionally, they showed intermediate resistance to gentamicin, ciprofloxacin, norfloxacin, and doxycycline, while they were susceptible to chloramphenicol and nitrofurantoin.

Regular exposure to antimicrobials facilitates the spread of slowly curable infections caused by *A. hydrophila*. In a previous study, Elkenany, [89] recovered 14.3% of *A. hydrophila* isolated from aquatic seafood organisms such as shrimp, crab, squid, and octopus in Egypt. They detected the *aer* and *hylA* genes in *A. hydrophila*. Additionally, they observed that *A. hydrophila* was resistant to amoxicillin, ceftriaxone, chloramphenicol, trimethoprim-sulfamethoxazole, and tetracycline. In a recent study, Lee, [90] detected antimicrobial resistance (AMR) genes such as *sul1*, in *A. hydrophila* in Norway. They also found *A. hydrophila* isolates resistant to erythromycin and florfenicol, with reduced susceptibility to oxolinic acid. Another study by Gharieb, [91] reported an overall 40.67% prevalence of *A. hydrophila* from *Tilapia nilotica* and *M. cephalus* in Egypt. They observed that *A. hydrophila* was resistant to carbenicillin and ampicillin, but susceptible to chloramphenicol, amikacin, ciprofloxacin, cefoxitin, cefotaxime, trimethoprim/sulfamethoxazole, and tetracycline. Moreover, Roges, [86] observed that *A. hydrophila* was highly resistant to cefoxitin, nalidixic acid, and tetracycline, with intermediate resistance to cefotaxime, imipenem, and ceftazidime. However, it was least resistant to amikacin, gentamicin, sulfamethoxazole-trimethoprim, ciprofloxacin, and nitrofurantoin. Similarly, Saleh, [82] observed that *A. hydrophila* was resistant to chloramphenicol, amikacin, and gentamicin, while highly susceptible to meropenem, ciprofloxacin, amoxicillin-clavulanic acid, and trimethoprim-sulfamethoxazole.

Virulence genes of pathogenic *A. hydrophila* cause serious histopathological effects in infected fish. In the current study, congestion (5.2%), hepatocyte necrosis (4.6%), granuloma formation (4.3%), and inflammation (5%) were observed in liver of infected fish. Epithelial hyperplasia (3.5%), lamellar fusion (3.3%), edema (3%), and the presence of *A. hydrophila* colonies (3.7%) in the gills. Fin erosion (6%), hemorrhage (6.2%), loss of fin rays (4.8%), and the presence of biofilms (3.5%) were observed in tail fins. Fibrosis (4%), abscess formation (3.7%), fatty degeneration (3.5%), and the infiltration of inflammatory cells (4.7%) were observed in spleen of infected fish. Histopathological effects of *A. hydrophila* infection were not studied in any previous study.

In the current study, we observed 100% and 97% similarity in the phylogenetic relationships of the *16S rRNA* and *gyrB* genes of *A. hydrophila*, respectively, among all the *A. hydrophila* strains isolated in this study, as well as with strains isolated in earlier studies. In a previous study, Wamala, [76] also analyzed the phylogenetic relationships through tree analysis of the *16S rRNA* and *gyrB* genes, revealing 100% and 99% similarity, respectively. Similarly, Esteve, [92] compared the phylogenetic relationships in Spain using the phylogenetic tree of the *16S rRNA* and *gyrB* genes of *A. hydrophila*, showing 100% similarity, consistent with our findings. Likewise, Li, [77] found 100% similarity in the phylogenetic relationships of the *16S rRNA* gene of *A. hydrophila* in China. In a recent study, Sani, [93] also observed 100% similarity in the phylogenetic relationships of the *16S rRNA* gene of *A. hydrophila* in Malaysia, which corroborated our results.

## Conclusion

Our examination of fish samples unveiled a concerning level of contamination with virulent and multidrug-resistant strains of *A. hydrophila*, highlighting the potential health risks associated with this contamination. The presence of pathogenic *A. hydrophila* results in significant histological changes in infected fish. The study underscores the importance of responsible antimicrobial use in aquaculture and the pressing need for effective strategies to curb the spread of virulence and antimicrobial resistance genes in *A. hydrophila*. Further research is imperative to delve into the mechanisms of virulence and resistance of *A. hydrophila* in fish.

## Supporting information

S1 Table Mean ± S.E of physico-chemical parameters of indus riverine system in Punjab. (DOCX)

S2 Table Phenotypic and biochemical characteristics of *A. hydrophila*. (DOCX)

S1 Raw data (XLSX)
