# Supplementary material for: Molecular characterization of Aeromonas hydrophila detected in Channa marulius and Sperata sarwari sampled from rivers of Punjab in Pakistan
Source: PLoS One. 2024 Mar 29;19(3):e0297979. doi: 10.1371/journal.pone.0297979 (PMC10980204; doi:10.1371/journal.pone.0297979)
Supplement: S1 Table — (DOCX) [file pone.0297979.s001.docx]

S1 Table. Mean± S.E of physico-chemical parameters of Indus riverine system in Punjab

| **Parameters** | **Head Baloki**  **(BL-H)** | **Head Trimmu**  **(TR-H)** | **Head Taunsa**  **(TA-H)** | **Head Chashma**  **(CH-H)** | ***p-*value** |
| --- | --- | --- | --- | --- | --- |
| **Air Temp. (°C)** | 28.54±2.24^a^ | 28.43±2.30^a^ | 34.79±2.65^a^ | 34.33±2.60^a^ | 0.121 |
| **Water Temp. (°C)** | 25.42±1.87^a^ | 26.62±1.48^a^ | 24.79±1.55^a^ | 22.98±2.00^a^ | 0.523 |
| **pH** | 7.72±0.13^b^ | 7.18±0.19^a^ | 8.07±0.17^bc^ | 8.23±0.08^c^ | 0.000 |
| **DO (ppm)** | 6.87±0.32^a^ | 8.07±0.24^b^ | 6.17±0.22^a^ | 8.13±0.19^b^ | 0.000 |
| **E.C (µs/cm)** | 265.53±8.07^a^ | 272.59±9.89^a^ | 269.99±9.15^a^ | 272.03±12.13^a^ | 0.957 |
| **TDS (mg/l)** | 170.03±5.17^b^ | 148.18±2.54^a^ | 169.65±3.36^b^ | 169.51±4.12^b^ | 0.000 |
| **Turbidity (FTU)** | 16.36±5.03^a^ | 15.89±4.77^a^ | 14.70±4.13^a^ | 12.44±2.76^a^ | 0.918 |
| **Visibility (cm)** | 87.33±9.08^b^ | 85.94±8.93^b^ | 68.33±6.55^ab^ | 55.58±6.64^a^ | 0.019 |
| **TH (mg/l)** | 144.33±3.48^b^ | 139.83±3.06^b^ | 123.83±5.66^a^ | 125.75±6.14^a^ | 0.007 |
| **Chlorides (mg/l)** | 28.25±1.06^a^ | 27.25±1.11^a^ | 30.75±1.21^a^ | 30.58±1.25^a^ | 0.101 |
| ***Sperata sarwari* (Singhari)** | | | | | |
| **Weight (g)** | 304.80±2.05^a^ | 303.80±1.85^a^ | 304.6000±2.03^a^ | 307.00±0.86^a^ | 0.625 |
| **Length (cm)** | 25.60±1.20^a^ | 27.40±1.25^a^ | 25.80±1.59^a^ | 27.00±0.83^a^ | 0.687 |
| ***Channa marulius* (Saul)** | | | | | |
| **Weight (g)** | 151.20±2.82^a^ | 163.00±5.14^ab^ | 170.40±6.14^b^ | 175.00±4.74^b^ | 0.017 |
| **Length (cm)** | 27.40±1.20^a^ | 30.80±1.59^ab^ | 32.72±1.77^b^ | 34.20±1.27^b^ | 0.028 |
