# Supplementary material for: Molecular characterization of Aeromonas hydrophila detected in Channa marulius and Sperata sarwari sampled from rivers of Punjab in Pakistan
Source: PLoS One. 2024 Mar 29;19(3):e0297979. doi: 10.1371/journal.pone.0297979 (PMC10980204; doi:10.1371/journal.pone.0297979)
Supplement: S2 Table — (DOCX) [file pone.0297979.s002.docx]

S2 Table. Phenotypic and biochemical characteristics of *A. hydrophila*

| **Phenotypic/Biochemical**  **Characteristics** | ***A. hydrophila*** |
| --- | --- |
| **Gram-Testing** | Gram-negative |
| **Aerobic or Anaerobic Nature** | Facultative anaerobe |
| **Cell morphology** | Rod-shaped |
| **Cell motility** | Motile |
| **Flagella** | Peritrichous flagella |
| **Shape of colony on TSA media plates** | Circular and smooth |
| **Diameter** | 1-4 mm |
| **Color of colony TSA media plates** | Grayish-white |
| **Catalase test** | Positive |
| **Oxidase test** | Positive |
| **Glucose test** | Positive |
| **Sucrose test** | Positive |
| **Lactose test** | Positive |
| **Indole production test** | Positive |
| **H_2_S production test** | Positive |
| **Urease test** | Positive |
